# Proteinase-activated receptor 2 modulates OA-related pain, cartilage and bone pathology

**DOI:** 10.1136/annrheumdis-2015-208268

**Published:** 2015-12-23

**Authors:** Carmen Huesa, Ana C Ortiz, Lynette Dunning, Laura McGavin, Louise Bennett, Kathryn McIntosh, Anne Crilly, Mariola Kurowska-Stolarska, Robin Plevin, Rob J van ‘t Hof, Andrew D Rowan, Iain B McInnes, Carl S Goodyear, John C Lockhart, William R Ferrell

**Affiliations:** 1 Institute of Biomedical & Environmental Health Research, University of the West of Scotland, Paisley, UK; 2 Institute of Infection, Immunity & Inflammation, University of Glasgow, Glasgow, UK; 3 Strathclyde Institute of Pharmacy and Biomedical Sciences, University of Strathclyde, Glasgow, UK; 4 Institute of Ageing and Chronic Diseases, University of Liverpool, Liverpool, UK; 5 Institute of Cellular Medicine, Newcastle University, Newcastle, UK

**Keywords:** Osteoarthritis, Synovitis, Chondrocytes, Inflammation

## Abstract

**Objective:**

Proteinase-activated receptor 2 (PAR2) deficiency protects against cartilage degradation in experimental osteoarthritis (OA). The wider impact of this pathway upon OA-associated pathologies such as osteophyte formation and pain is unknown. Herein, we investigated early temporal bone and cartilage changes in experimental OA in order to further elucidate the role of PAR2 in OA pathogenesis.

**Methods:**

OA was induced in wild-type (WT) and PAR2-deficient (PAR2^−/−^) mice by destabilisation of the medial meniscus (DMM). Inflammation, cartilage degradation and bone changes were monitored using histology and microCT. In gene rescue experiments, PAR2^−/−^ mice were intra-articularly injected with human PAR2 (hPAR2)-expressing adenovirus. Dynamic weight bearing was used as a surrogate of OA-related pain.

**Results:**

Osteophytes formed within 7 days post-DMM in WT mice but osteosclerosis was only evident from 14 days post induction. Importantly, PAR2 was expressed in the proliferative/hypertrophic chondrocytes present within osteophytes. In PAR2^−/−^ mice, osteophytes developed significantly less frequently but, when present, were smaller and of greater density; no osteosclerosis was observed in these mice up to day 28. The pattern of weight bearing was altered in PAR2^−/−^ mice, suggesting reduced pain perception. The expression of hPAR2 in PAR2^−/−^ mice recapitulated osteophyte formation and cartilage damage similar to that observed in WT mice. However, osteosclerosis was absent, consistent with lack of hPAR2 expression in subchondral bone.

**Conclusions:**

This study clearly demonstrates PAR2 plays a critical role, via chondrocytes, in osteophyte development and subchondral bone changes, which occur prior to PAR2-mediated cartilage damage. The latter likely occurs independently of OA-related bone changes.

## Introduction

Osteoarthritis (OA) is the most common musculoskeletal disorder, affecting up to 80% of people aged >65 years. Dysregulated proteolysis occurs in OA, but there are no clinically effective matrix metalloproteinase inhibitors. This has led to a search for upstream regulatory and therapeutically tractable pathways that drive downstream pathological processes. Proteinase-activated receptor 2 (PAR2) is activated by specific serine proteases (eg, matriptase^1^), which mediates signalling and internalisation of the receptor complex. Recognised to have a pro-inflammatory role in the musculoskeletal system,^2^ ^3^ recent work suggests that PAR2 also plays a role in OA.

We previously demonstrated in experimental OA generated by destabilisation of the medial meniscus (DMM) that PAR2-deficient mice (PAR2^−/−^) were significantly protected from cartilage damage and osteosclerosis,^4^ subsequently confirmed by others.^5^ ^6^ While these studies showed reduced subchondral bone sclerosis in PAR2^−/−^ mice, its role in the early stages of disease, particularly osteophyte development, has not been comprehensively investigated. The principal aim of the present study was to examine the role of PAR2 in early disease and in osteophyte formation using micro-CT (μCT). We also characterised whether the pathogenic phenotype observed in wild-type (WT) mice following DMM could be re-established in PAR2^−/−^ mice following transfection of the knee with an adenoviral vector expressing PAR2.

## Methods

### Animals

Experiments were performed on adult (25–30 g) male PAR2^−/−^ mice (C57BL/6J backcrossed to at least 10 generations), genetically modified as previously described,^2^ with WT (PAR2^+/+^) littermates as controls. All procedures were in accordance with Home Office regulations.

### Induction of OA

As previously described,^4^ medial compartment OA was induced by DMM following transection of the left medial meniscotibial ligament under aseptic conditions. Buprenorphine (Vetergesic; 30 μg intraperitoneally) was administered postoperatively and animals maintained for 3, 7, 14 and 28 days, with knee joints subsequently harvested for μCT and histology.

### PAR2 transfection

The left knee joints of five PAR2^−/−^ mice were injected with an adeno-associated viral vector (serotype 2/5), which included a cytomegalovirus promoter for human PAR2 (hPAR2) and a C-terminal mCherry tag (Penn State, USA). Five other mice acted as controls following administration of AAV2/5 CMV Luciferase. The latter also enabled assessment of the efficiency of transfection and longevity of the virus in the joint, using IVIS technology (see online supplementary methods). Three days after injection, DMM was performed with mice sacrificed after 4 weeks.

### MicroCT

Knee joints were fixed in 4% paraformaldehyde solution for 24 h and subsequently stored in 70% EtOH, then analysed by μCT to examine the calcified tissues using Skyscan 1272 (Bruker, Belgium; 0.5 aluminium filter, 50 kV, 200 µA, voxel size 4.57 μm, 0.5° rotation angle). Scans were reconstructed in NRecon software (Bruker, Belgium), with stacks analysed as follows: (1) osteophytes were identified in three-dimensional reconstructions of the stacks as detailed (see online supplementary methods) and (2) subchondral bone was analysed by selecting a volume of interest, delineating the trabecular structure within the tibial epiphysis. Parameters were assessed as a medial/lateral ratio and compared with the contralateral leg using a paired t test.

### Assessment of cartilage damage

Histological analysis of progression and severity of cartilage damage was undertaken on joints previously scanned, then decalcified (Formical 2000; Decal Chemical, New York, USA) overnight. Joints were embedded in paraffin wax and coronal sections (6 μm) cut then stained with haematoxylin, safranin-O/fast green. Using a validated scoring system^7^ ranging from 0 (normal) to 6 (>80% loss of cartilage), the tibial quadrant in 8–10 sections from each mouse was graded by two scorers blinded to the specimens, with scores averaged. There was good agreement between scorers with intraclass correlation coefficient of 0.9 (95% CI 0.72 to 0.97), the mean difference in score being 0.12 (95% CI −0.39 to 0.63).

### Immunohistochemistry

Following decalcification, sections were deparaffinised, rehydrated and probed with selected antibodies. Anti-SOX9 monoclonal antibody (Millipore, UK), anti-F4/80 (Abcam, UK), anti-mCherry (Abcam) and anti-Runx2 (Insight, UK) were used as well as SAM11 (Santa Cruz Biotech, USA). Primary antibodies were detected using the Vectastain ABC kit with a secondary pan-specific biotinylated antibody (Vectorlabs, UK), visualised using diaminobenzidine (DAB; Vectorlabs) and counterstained with haematoxylin (Sigma, UK).

### Assessment of synovitis

This was assessed initially using IVIS 200 imaging (Xenogen, California, USA) using the myeloperoxidase/luminol system and scanned at various time points following DMM. In addition, synovitis was assessed histologically using a recently developed scoring system.^6^ This was modified to focus only on pannus formation, synovial membrane thickening and subsynovial hyperplasia (see online supplementary table S1). Agreement between scorers was good with intraclass correlation coefficient of 0.88 (95% CI 0.65 to 0.95), the mean difference in score being 0.1 (95% CI −0.46 to 0.67).

### Dynamic weight bearing

As an indirect indicator of pain, limb weight bearing was assessed in mice before and after surgery using the BioSeb chamber (BioSeb, Marseilles, France). Animals were individually recorded for 5 min, of which a minimum of 2 min was subsequently validated and analysed. The parameters examined were the load on the front paws and the per cent of time spent on the front paws.

### Statistical analysis

Data were tested for normality (Sigmastat 2.03; SPSS) and expressed in graphs as mean±SEM with comparisons by one-way or two-way repeated-measures analysis of variance (ANOVA) and multiple comparisons using Bonferroni correction.

## Results

### Osteophyte development

**Figure 1:**
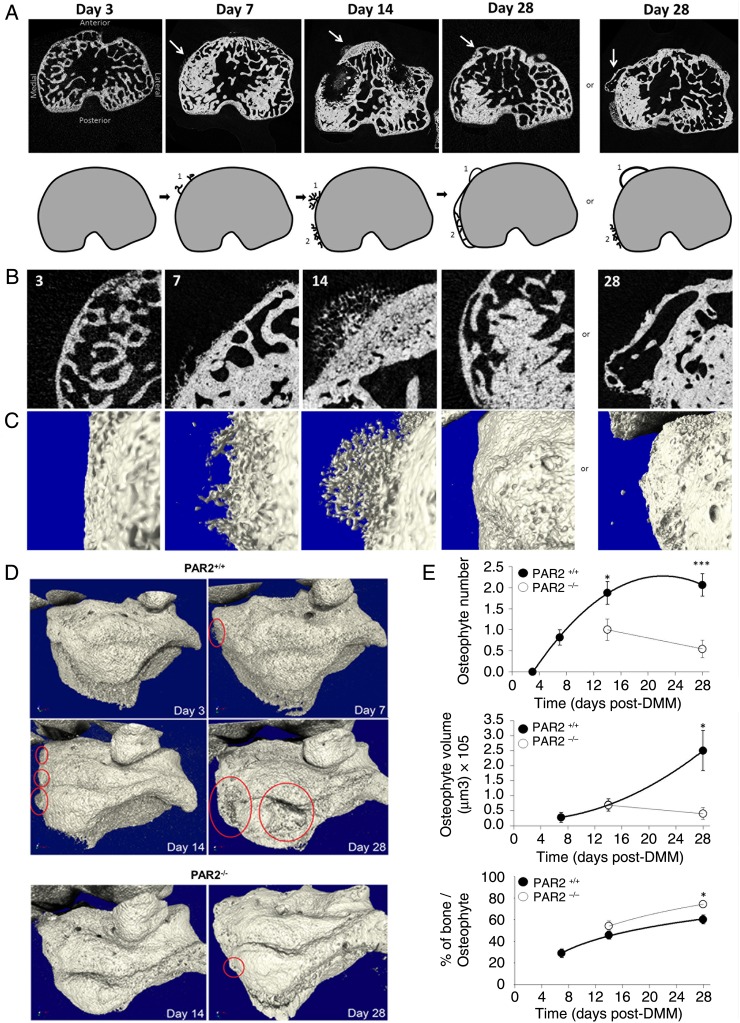
Time course of osteophyte development (A) microCT (μCT) images showing time course of developing osteophytes (arrows) following destabilisation of the medial meniscus (DMM) in wild-type (WT) mice. Cartoons depict development of osteophytes (1) and the expansion of the subchondral plate (2). By day 28, the expansion of the subchondral bone is complete, yet protruding osteophytes remain a prominent feature. (B) Two-dimensional μCT magnification of the medial anterior side of the subchondral bone and (C) their corresponding three-dimensional images. (D) μCT images showing representative examples of osteophytes (circled) at different time points in WT (PAR2^+/+^) and proteinase-activated receptor 2 (PAR2)-deficient mice (PAR2^−/−^). (E) Quantitative data showing reduced osteophyte number and volume but elevated bone content in PAR2^−/−^ mice compared with WT littermates. *p<0.05; ***p<0.001 comparing PAR2^+/+^ to PAR2^−/−^ mice. n=10–12.

Osteophytes were undetectable in sham-operated mice. Development of osteophytes in WT mice was observed from 7 days post DMM (figure 1A), which increased in size and number over time (figure 1D, E). Initially arboreal in appearance (day 14, figure 1B, C), an additional layer of bone formed by day 28 (figure 1A–C). However, large protruding osteophytes were still evident in 12/13 WT mice at that time point (figure 1A, B). While PAR2^−/−^ mice similarly developed an additional bone layer (see online supplementary figure S1), only 5/11 exhibited osteophytes at day 28. If present, these were smaller and did not increase in size with time (figure 1D, E). The composition of osteophytes in PAR2^−/−^ mice differed from WT, with increased bone density even at the point of first assessment (figure 1E).

### Osteophyte cell phenotype

**Figure 2:**
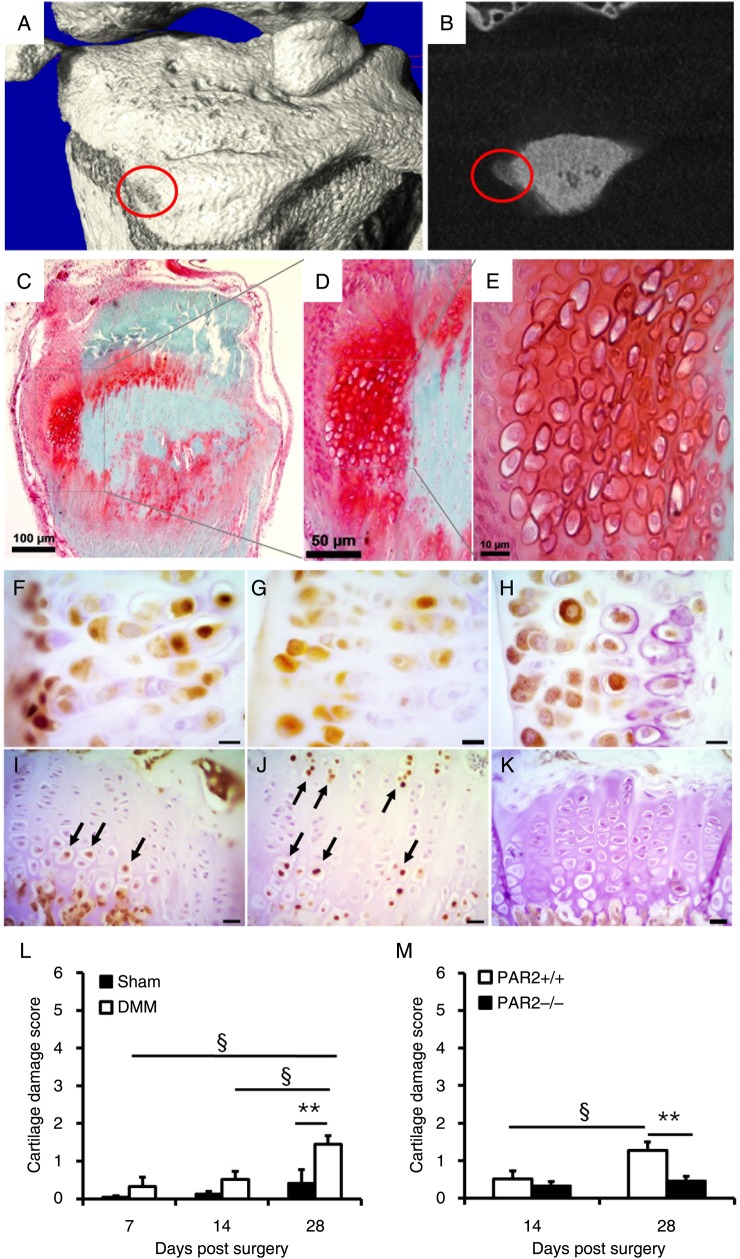
Cell phenotype in developing osteophytes and articular cartilage damage following destabilisation of the medial meniscus (DMM) three-dimensional reconstruction of μCT data set (A) and cross-sectional image (B) showing the presence of an osteophyte (circled) in a wild-type (WT) mouse 14 days following DMM. This was confirmed by histological appearance of the same osteophyte (C) and at higher magnification (D and E). Cells in osteophytes have a chondrocytic appearance and express SOX9 (F), Runx2 (G) and proteinase-activated receptor 2 (PAR2) (H). Cells (arrowed) in the growth plate express SOX9 (I) and Runx2 (J) but not PAR2 (K). Scale bars F, G, H=10 μm; I, J, K=20 μm. (L) Averaged cartilage damage scores at different time points following DMM compared with sham-operated WT mice. **p<0.005, DMM versus sham; §p<0.02 DMM comparison at different time points. n=4–8. (M) Cartilage damage scores following DMM comparing PAR2^+/+^ to PAR2^−/−^ mice. **p<0.005; §p<0.02. n=8/group.

Mineralised osteophytes identified by µCT (figure 2A, B) were histologically characterised as being of a chondrocytic phenotype (figure 2C–E). Subsequent immunohistochemical analysis revealed SOX9 and Runx-2 expression, confirming that these cells were chondrocytes with a proliferative/hypertrophic phenotype^8^ (figure 2F, G). These prehypertrophic chondrocytes also strongly expressed PAR2 (figure 2H), but this appeared to be pathological because, although cells in the growth plate expressed both SOX9 (figure 2I) and Runx2 (figure 2J), PAR2 was absent in growth plate chondrocytes (figure 2K).

### Cartilage damage following DMM

Mean cartilage damage scores were temporally compared following DMM or sham operation in WT mice. There was no observed cartilage damage 3 days following DMM or sham operation (data not shown), and while a tendency to increased scores was observed at 7 and 14 days, these did not differ significantly compared with sham. However, by day 28 structural damage was evident in DMM mice and scores differed significantly from the earlier DMM time points (figure 2L). For the sham-operated group, there was no significant difference in scores across time points.

A comparison of cartilage damage scores following DMM showed no difference between WT and PAR2^−/−^ mice at day 14, but by day 28, these groups differed significantly (figure 2M), with scores in the PAR2^−/−^ mice approximately half of those in the WT mice. The scores in the PAR2^−/−^ group did not significantly increase between the day 14 and 28 time points, nor was there any difference compared with sham (p=0.43 and 0.13, respectively).

### Subchondral bone changes and weight bearing following DMM

**Figure 3:**
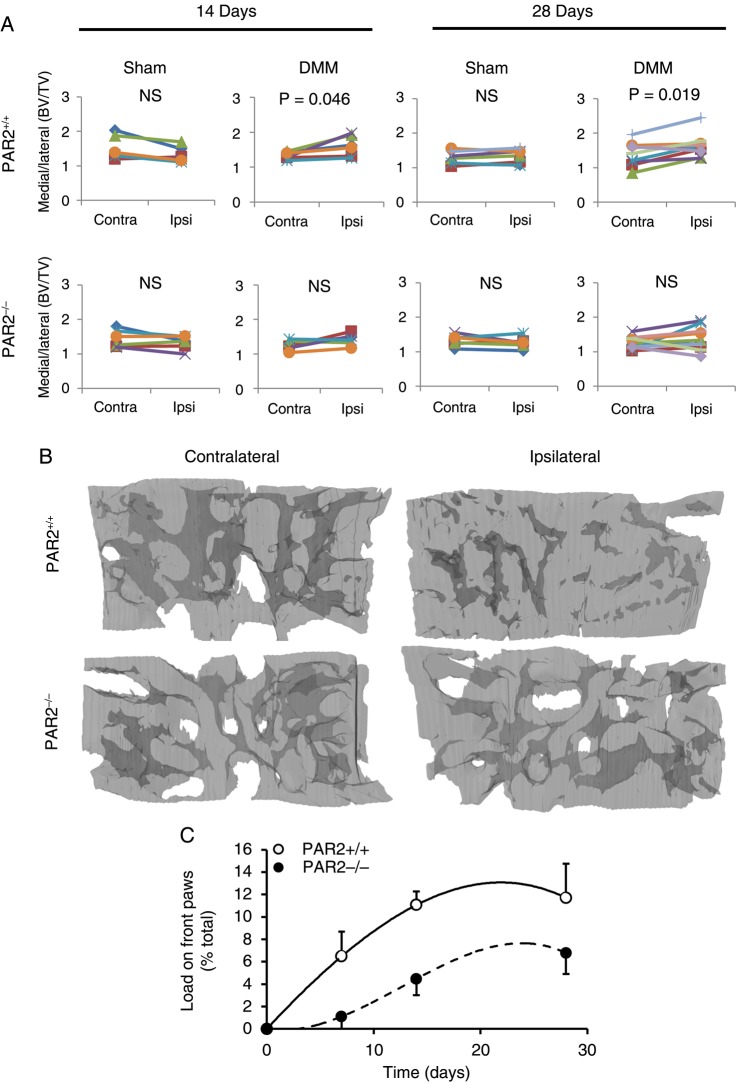
Subchondral bone changes following destabilisation of the medial meniscus (DMM). (A) Comparison of bone volume over tissue volume (BV/TV) changes 14 and 28 days following sham or DMM operation in wild-type (WT) or proteinase-activated receptor 2-deficient (PAR2^−/−^) mice. Significance values refer to differences in the BV/TV medial tibial to lateral tibial ratio comparing the contralateral unoperated knee (Contra) to the operated (Ipsi) knee. Each line refers to an individual mouse. (n=6–9). (B) Representative three-dimensional models of contralateral medial tibial subchondral trabecular region compared with their ipsilateral (operated) counterpart 28 days after DMM showing increased subchondral bone density in the operated WT knee. Bone is shaded light grey and made translucent to allow visualisation of bone marrow spaces (dark grey). (C) Time course of dynamic weight bearing, measuring the load on the front paws normalised to pre-surgical load in DMM-operated WT and PAR2^−/−^ mice. n=5–6.

µCT analysis of the subchondral trabecular bone in WT mice showed no significant changes in the bone volume over tissue volume medial to lateral ratio at days 3 and 7 post DMM. However, by day 14 post-DMM surgery, the operated (ipsilateral) knee in WT mice significantly increased compared with the contralateral knee, which was maintained through day 28 (figure 3A). In PAR2^−/−^ mice, there was no significant difference between contralateral and ipsilateral knees following DMM surgery at days 14 and 28. The difference between WT and PAR2^−/−^ mice was reflected in the greater medial tibial subchondral bone density in the WT mice (figure 3B).

To assess OA-related pain, measurement of weight bearing 4 weeks post DMM showed a difference over time between groups, WT mice placing significantly more load on the front paws than PAR2^−/−^ mice (p=0.034, two-way ANOVA; figure 3C). In sham-operated mice, there was no significant difference between genotypes (data not shown).

### Synovitis following DMM

Although not considered to be an inflammatory OA model,^9^ a recent investigation using a novel scoring system observed low level of synovitis following DMM compared with sham controls.^6^ However, synovitis scores did not differ between WT and PAR2^−/−^ mice. Given that PAR2 is recognised to be pro-inflammatory,^10^ combined with substantial reduction of adjuvant-induced monoarthritis in PAR2^−/−^ mice,^2^ we assessed synovitis in the current study.

**Figure 4:**
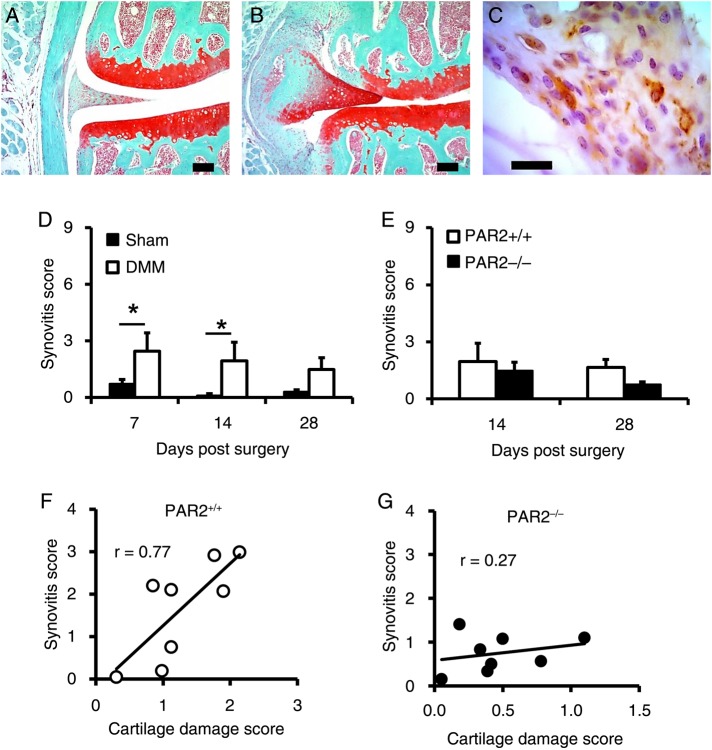
Time course of synovitis following destabilisation of the medial meniscus (DMM). Histological appearance of the medial compartment of the knee joint 28 days following sham operation (A) or DMM (B) in proteinase-activated receptor 2-deficient (PAR2^+/+^) mice, showing thickening of the medial collateral ligament and cellular hyperplasia in the latter. Scale bar=100 μm. (C) Immunohistochemical analysis of the synovium 28 days post DMM in a PAR2^+/+^ mouse showing some cells staining for the macrophage marker F4/80. Scale bar=20 μm. (D) Synovitis scores in wild-type (WT) mice at different time points following DMM or sham operation. n=4–8. *p<0.05, sham compared with DMM. (E) Synovitis scores at 14 and 28 days post DMM comparing PAR2^+/+^ to PAR2^−/−^ mice. Synovitis and cartilage damage scores at day 28 are significantly correlated for WT (F) but not PAR2^−/−^ (G) mice. n=8/group.

Although myeloperoxidase activity, indicative of synovitis, was detectable using IVIS imaging in an adjuvant monoarthritis model (positive control), no sustained signal was observed following DMM (see online supplementary figure S2). However, using our modified synovitis histological scoring system, we found evidence of synovitis following DMM in WT mice compared with sham (figure 4A, B). Macrophage-like F4/80^+^ cells were detected in synovia 7 days following DMM (figure 4C). Compared with sham-operated, synovitis scores following DMM were significantly higher in WT at 7 and 14, but not 28 days (figure 4D). Synovitis scores following DMM were similar in WT and PAR2^−/−^ mice at day 14 postoperatively (figure 4E), and although decreased at day 28 in PAR2^−/−^ mice, this was not significant (p=0.057). Nevertheless, while there was a strong relationship between cartilage damage and synovitis scores in WT mice at day 28 (r^2^=0.59, p=0.026; figure 4F), there was no comparable correlation (r^2^=0.07, p=0.2) for PAR2^−/−^ mice (figure 4G).

### Restoration of pathogenic phenotype

**Figure 5:**
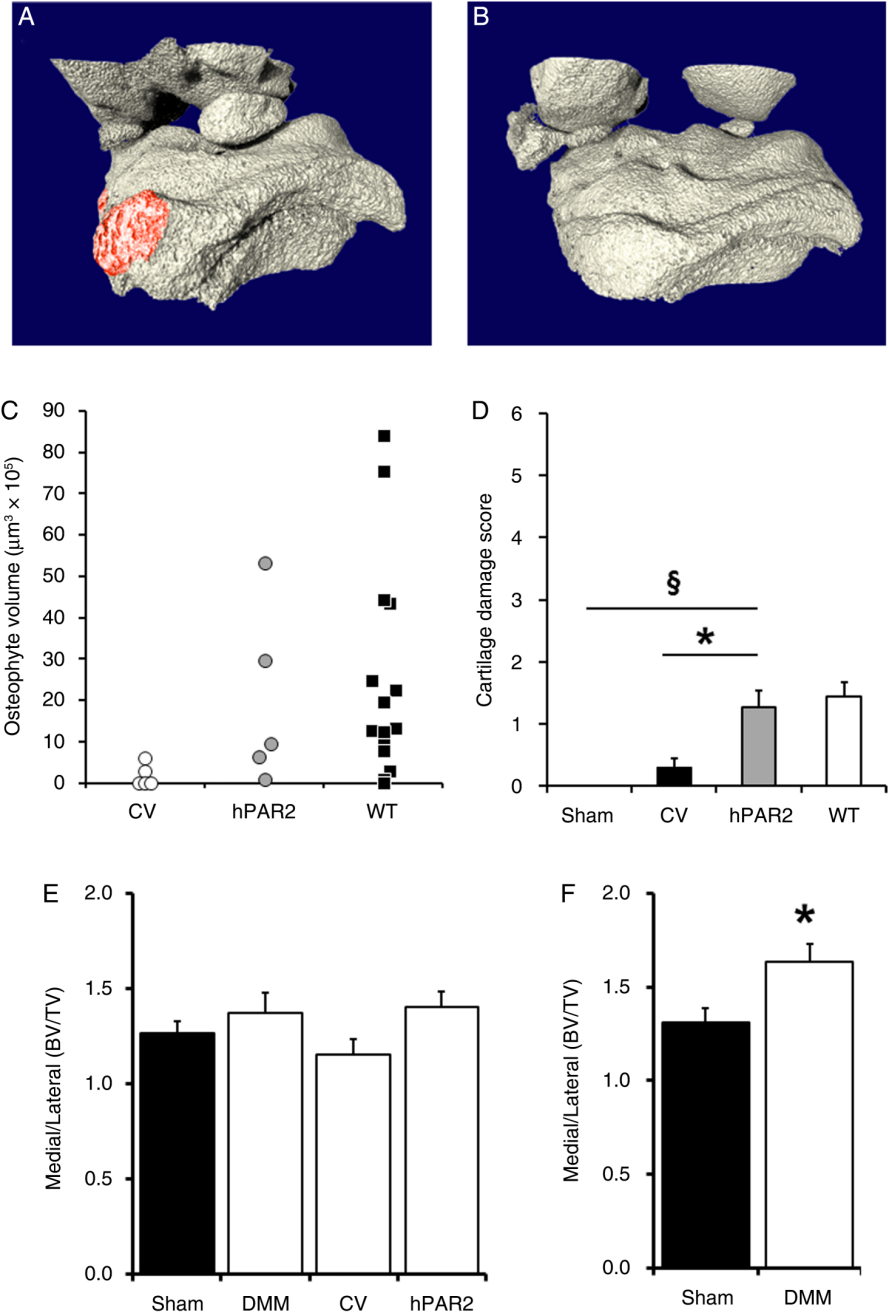
Restoration of pathogenic phenotype. Examples of 3D microCT images taken 4 weeks following destabilisation of the medial meniscus (DMM) from proteinase-activated receptor 2-deficient (PAR2^−/−^) mice administered either (A) the AAV2/5 adenoviral vector containing human PAR2 (hPAR2) (osteophytes highlighted in red) or (B) luciferase control. (C) Osteophyte volume was greater in the hPAR2-treated mice compared with mice administered control vector (CV), albeit with large variability. (D) Cartilage damage scores in PAR2^−/−^ mice administered hPAR2 were greater than either sham-operated or CV-administered PAR2^−/−^ mice. Bone volume over tissue volume (BV/TV) medial to lateral ratio of the ipsilateral leg showed no significant differences in all the PAR2^−/−^ groups (E) while this was significantly greater in wild-type (WT) DMM mice compared with sham (F). *p<0.05; §p<0.01. n=5.

As deletion^4–6^ or inhibition^4^ of PAR2 confers protection from OA in the DMM model, we investigated whether intra-articular injection of a viral vector expressing mCherry-tagged hPAR2 in PAR2^−/−^ mice restores the pathogenic phenotype. A parallel group of PAR2^−/−^ mice received an intra-articular injection of an AAV2/5 control vector expressing the luciferase gene. A strong luciferase signal was observed in mice up to 28 days following DMM (see online supplementary figure S3) confirming transfection longevity. Using an AAV-2/5 lacZ vector, β-galactosidase expression in articular chondrocytes was evident 3 weeks following injection and this was further confirmed by the presence of mCherry staining in chondrocytes and the synovial membrane 28 days following DMM in hPAR2 but not control vector-transfected mice (see online supplementary figure S3). Interestingly, there was no mCherry staining in the subchondral bone of hPAR2-transfected mice. In all cases (5/5), hPAR2-transfected mice developed osteophytes (figure 5A) consistent with those observed in WT mice, whereas in the control group, only 2/5 developed small osteophytes (figure 5B, C). Similarly, cartilage damage scores were significantly lower in the control group compared with the hPAR2-transfected group, and the former did not differ from sham-operated PAR2^−/−^ mice (figure 5D). Comparisons with WT did not show any differences (see online supplementary figure S4). Following DMM in PAR2^−/−^ mice, cartilage damage was present in the hPAR2-transfected group despite no significant difference in subchondral bone sclerosis compared with control vector, non-transfected or sham-operated PAR2^−/−^ mice (figure 5E), unlike WT mice, which showed significantly greater bone sclerosis compared with sham (figure 5F).

## Discussion

While others have reported bone changes in the DMM model using µCT,^11^ ^12^ the present study is the first to investigate early osteophyte development in this model and characterise the temporal role of PAR2 in osteophyte emergence. An important observation was that WT mice developed osteophytes within 7 days from induction, which continued to enlarge over time. We hypothesise that osteophytes develop to expand the tibial plateau area, the latter having been proposed as a response mechanism to altered biomechanical loading in OA.^13^ ^14^ Expansion was visualised by day 28 as a second layer of bone on the medial aspect revealed by transaxial and coronal µCT sections. While a comparable process occurs in WT and PAR2^−/−^ mice, and therefore not PAR2-dependent, this appears dysregulated in the WT as evidenced by a high incidence of large mineralised osteophytes by day 28. Although osteophytes were detectable in both WT and <50% of PAR2^−/−^ mice, there were clear differences in incidence, size, mineralisation and subsequent enlargement. This contrasts with a recent histological analysis, which found no differences in osteophyte maturity and size at 4 weeks post DMM.^6^ This may reflect the different analysis methods used, with µCT providing a more quantitative measure of pathology. The differences observed in osteophyte parameters between genotypes suggest a role for PAR2 in OA-related osteophyte maturation. This is supported by the finding of PAR2 expression in proliferative cells within osteophytes, identified immunohistochemically as being derived from the chondrocyte lineage, the latter consistent with previous observations.^15^ Interestingly, although chondrocyte markers SOX9 and Runx2 are present in the growth plate, PAR2 is absent, which suggests that its presence in osteophytes is pathological and could explain why PAR2^−/−^ mice do not exhibit an abnormal growth phenotype. Osteophyte formation has parallels with callus formation, and it is interesting to note that callus morphology is altered in PAR2^−/−^ mice.^16^ Osteophyte formation is clearly PAR2-dependent, and a preliminary observation that serum levels of let-7e were lower in naive PAR2^−/−^ mice compared with WT littermates (see online supplementary figure S5) suggests involvement of let-7e in the pathway.

This study temporally characterised the onset of pathological changes in DMM, demonstrating that observable subchondral bone changes preceded cartilage damage in this model. This may reflect differences in the dynamic responsiveness of skeletal versus cartilaginous tissues.^17^ Consistent with previous histological studies,^4–6^ µCT analysis of the joint demonstrated osteosclerosis was clearly evident following DMM in WT but not PAR2^−/−^ mice, suggesting a role for this receptor in mechanosensing and/or mechanotransduction. The time differential between bone and cartilage changes appears to support the hypothesis that osteosclerosis following DMM may alter loading, resulting in direct cartilage damage.^6^ This implies osteosclerosis is a necessary prerequisite for cartilage damage. Indeed, cartilage damage and subchondral bone thickening in this OA model were found to be significantly correlated in a recent study.^6^ However, our observation of significant cartilage damage without associated osteosclerosis in hPAR2-transfected mice does not support this hypothesis. Furthermore, inhibiting sclerostin in a rat injury model of OA resulted in a significant increase in epiphyseal bone density but no difference in cartilage damage.^18^ This leads us to conclude that cartilage damage in the DMM model is mediated via PAR2, independent of osteosclerosis.

A novel approach was use of dynamic weight bearing (DWB) as a surrogate assessment of OA-related pain. The pattern of weight bearing across the limbs in the PAR2^−/−^ mouse clearly differed from the WT, consistent with reduced nociception in the former. This is consistent with PAR2^−/−^ mice having diminished hyperalgesia,^19^ and impairment of hindlimb weight bearing in WT rodents following knee joint injection of a PAR2 agonist.^20^ DWB, thus, offers a valuable non-invasive method for assessment of pain in murine models of arthritis.

Recently, analysis of cartilage damage revealed little change between WT and PAR2^−/−^ mice 7 days post-DMM induction,^6^ so we investigated whether damage is evident at day 14. In WT, there was no significant change until day 28 compared with sham-operated, hence no difference was observed compared with PAR2^−/−^ mice at day 14. Confirming our earlier study,^4^ PAR2 deletion protects against cartilage damage 28 days post DMM, consistent with other investigations of PAR2 in this OA model.^5^ ^6^ Notably, this is restricted to PAR2 as PAR1 deletion does not confer such protection,^6^ underlining the specificity of PAR2 in OA pathogenesis.

There is increasing recognition of the role of synovial inflammation in OA pathogenesis as it is linked to the severity of knee OA,^21^ and synovitis is detectable by MRI in 90% of patients with knee OA.^22^ In humans, PAR2 is associated with synovitis in OA with the degree of synovitis and PAR2 expression being strongly correlated.^23^ Thus, PAR2 is a likely contributor to synovitis. Although the DMM model is considered non-inflammatory,^9^ this has been challenged recently^6^ where histological evidence of synovitis was detected. In this previous study, synovitis scores at early time points (3–14 days) did not differ between sham-operated and DMM groups, although these diverged later. However, there was no difference in synovitis scores between WT and PAR2^−/−^ mice at 28 days post induction of DMM.^6^ Conversely, we herein found that WT scores differed significantly between sham-operated and DMM groups at days 7 and 14, with a clear trend for scores to be lower in PAR2^−/−^ mice. This discrepancy, particularly in sham-operated mice, may reflect the plane of section: we took coronal rather than sagittal sections as used by Jackson *et al*,^6^ which included regions of the knee exposed during surgery. Sagittal sectioning would include regions of wound healing, presumably similar in both sham and DMM WT groups. Indeed, the incision site was included as Jackson *et al*^6^ consider inflammation associated with surgery a major contributor to synovitis and the pathophysiology of joint disease, particularly in early stages postoperatively (Little CB, personal communication). This indicates a potential limitation of the DMM model as it involves injury with consequent inflammation. Further work is required to determine the causative role (if any) of synovitis in OA pathogenesis and how PAR2 influences OA in DMM via its pro-inflammatory actions.

A key finding was that in PAR2^−/−^ mice the OA phenotype could be re-established by intra-articular administration of hPAR2 using an adenoviral vector. Thus, PAR2 transfection promotes cartilage degradation, confirming PAR2's pathogenic role in DMM. More surprisingly, osteophyte formation was also affected, some hPAR2-transfected mice developing very large osteophytes. Our data suggest that intra-articular transfection will likely only introduce PAR2 into cells in the immediate vicinity, indicating that in this context PAR2 may be directly affecting chondrocyte proliferation/hypertrophy, leading to osteophyte formation (figure 2). We also believe that transfection-induced expression of hPAR2 in osteoblasts is unlikely, given the absence of mCherry staining in subchondral bone (see online supplementary figure S3), possibly explaining absence of osteosclerosis in hPAR2-transfected mice following DMM. This in turn may indicate that some pathogenic features in DMM are driven by PAR2 mechanisms affecting chondrocytes. This view differs from that proposed recently where interleukin-1α-induced degradation in cartilage explant cultures from PAR2^−/−^ mice was not inhibited, leading the authors to suggest that extra-cartilaginous mechanisms may drive pathogenesis.^6^

Our central conclusion is that OA-related changes in bone and cartilage are dependent on, and therefore mediated by, PAR2, accelerating the pathogenic phenotype. Moreover, our temporal characterisation of early changes in OA demonstrates that although bone changes precede, they do not necessarily drive cartilage damage, which appears to occur independently, indicated by lack of osteosclerosis in hPAR2-transfected PAR2^−/−^ mice. This challenges a long-standing view that increased stiffness of subchondral bone leads to overlying cartilage lesions.^24^ The protection offered by PAR2-deficiency may be related to the role of this receptor in driving pathological chondrocyte differentiation/proliferation. Therapeutically, targeting PAR2 may offer value not only in abrogating OA structural changes, but also alleviating arthritic pain.

## Supplementary Material

Web supplement

Web supplement

Web figures

Web table
