# Supplementary material for: Proteinase-activated receptor 2 modulates OA-related pain, cartilage and bone pathology
Source: Ann Rheum Dis. 2015 Dec 23;75(11):1989–97. doi: 10.1136/annrheumdis-2015-208268 (PMC5099200; doi:10.1136/annrheumdis-2015-208268)
Supplement: Web supplement [file annrheumdis-2015-208268-s2.pdf]

Supplemental Methods

Micro computed tomography

Image stacks were analysed as follows: 1) Osteophytes were identified in three dimensional reconstructions of the stacks as using CTvol software (Bruker, Belgium) and quantified. Osteophyte volume was measured by manually delineating the edge of the largest osteophyte protruding from the subchondral plate in each sample as the region of interest (ROI) to be analysed and then quantified for total volume and bone volume with CTan software (Bruker, Belgium). All analysis was performed with a voxel size of 4.57 μm, and while the default resolution for scanning was 4.57μm, 2μm resolution was used for scans in which we sought to enhance osteophyte visualisation (Figure 1). 2) Subchondral bone was analysed by selecting a volume of interest (VOI) of 0.5 x 0.9 x 0.9 mm^3 in the medial and lateral regions of the tibial plateau. Two ROIs (medial and lateral) delineating the trabecular structure within the tibial epiphysis were selected in the 2D coronal view of the stack to analyse the bone volume and microarchitecture. Parameters were assessed as a medial/lateral ratio and compared to the contralateral leg using a paired t-test.

IVIS Methodology

PAR2-transfection: The longevity and efficiency of transfection was assessed in control mice (AAV2/5 CMV Luciferase) by pre-dosing with D-Luciferin (Perkin Elmer, Buckinghamshire, UK) at 150 mg/kg and sampling every 5 min. Luminescence image acquisition (1 second, f-stop 1 and small [setting 4] binning) was performed using a Xenogen IVIS System (Caliper Life Sciences) 15 min after injection. Living Image software (Caliper Life Sciences) was used to analyse data with radiance (photons/sec/cm^2/sr) measured. Regions of interest (ROI) were drawn around each knee joint and total flux calculated. For both hPAR2 and luciferase control, approximately 1.3 x10^{10} genome copies were administered by intra-articular injection. Three days after injection, DMM was performed with mice sacrificed after 4 weeks.

Assessment of synovitis: To assess myeloperoxidase activity, Luminol sodium salt (Sigma-Aldrich, Dorset, UK) was administered I.P. at 200 mg/kg with IVIS parameters of: acquisition time of 5 min, f-stop 1 and medium binning (setting 8).

miRNA Extraction and Quantification

miRNAs were extracted from serum with miRNeasy mini kit (Qiagen, Manchester, UK). Plasma (50μl) was spiked with 1 x 10^6 copies/μl of the *C.Elegans* spike-in control (Qiagen,

Manchester, UK) and chloroform phase separation performed. The upper aqueous phase was added to the spin-column and the membrane was washed with RWT, followed by RPE buffer and finally 80% ethanol. RNA was eluted in RNase-free water. cDNA was synthesised with 10µl of the eluted RNA following the Miscript II RT kit protocol (Qiagen, Manchester, UK). qPCR was performed with qiagen primers for miRNA140, Let7e, and *C.Elegans* miRNA 39. miScript SYBR green (Qiagen, Manchester, UK) was used with all samples. Data are presented as $2^{-\Delta\Delta Ct}$. First, Ct values for "spike in" control (miRNA 39) were subtracted from Ct values of miR of interest (ΔCt). Then, mean ΔCt for WT were used as a calibrator and subtracted from all the ΔCt values (ΔΔCt).

1. Papaioannou G, Inloes JB, Nakamura Y, et al. let-7 and miR-140 microRNAs coordinately regulate skeletal development. PNAS 2013;110:E3291-300.
