# Supplementary material for: Proteinase-activated receptor 2 modulates OA-related pain, cartilage and bone pathology
Source: Ann Rheum Dis. 2015 Dec 23;75(11):1989–97. doi: 10.1136/annrheumdis-2015-208268 (PMC5099200; doi:10.1136/annrheumdis-2015-208268)
Supplement: Web figures [file annrheumdis-2015-208268-s3.pdf]

Supplemental Figures

Supplemental Figure 1

Cross-sectional images of WT and PAR2$^{-/-}$ mice at days 14 and 28 following DMM, showing development of a double layer of bone on the medial side of the joint. Note absence of osteophytes in PAR2$^{-/-}$ mice compared to WT (arrows).

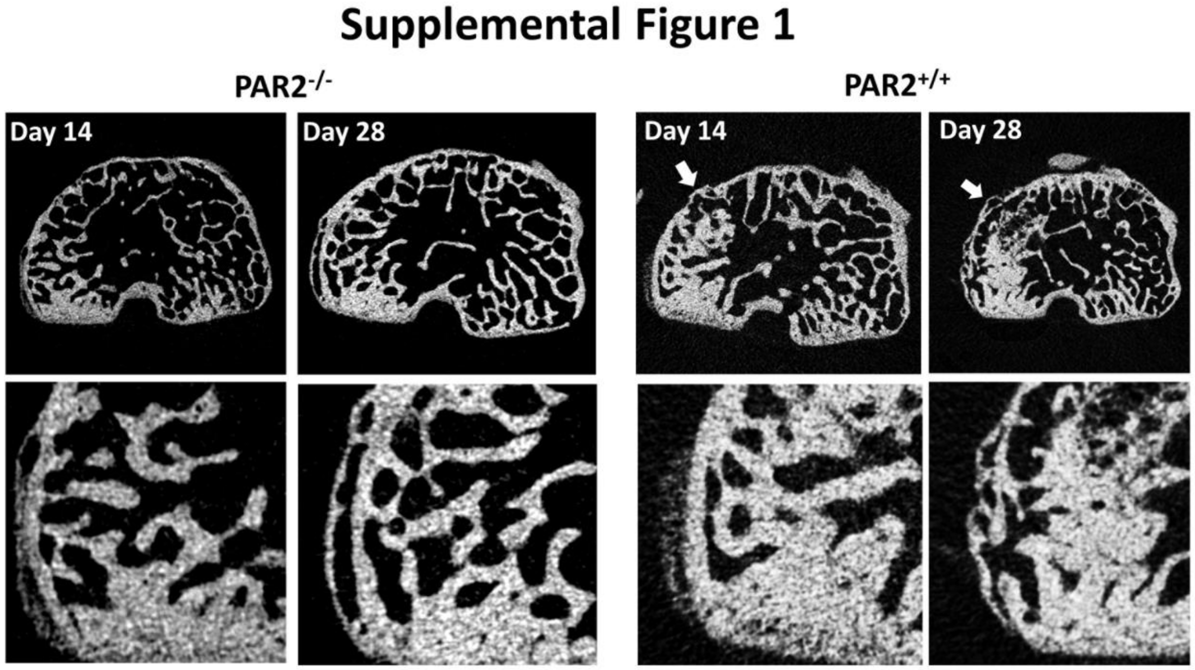

Supplemental Figure 2

The Xenogene IVIS 200 system was used to detect the time course of myeloperoxidase activity following luminol administration (200mg/kg i.p.) in WT mice following induction of adjuvant mono-arthritis by administration of Freund's complete adjuvant (FCA) compared to mice undergoing DMM surgery. **A**: scans from individual mice at weekly intervals show progressive rise in signal following FCA induction but minor changes following DMM. **B**: quantitative data from 5 mice in each group confirming the progressive increase in myeloperoxidase activity in the FCA model of arthritis but only small changes following DMM. Interestingly, the highest signal in the DMM group occurred at day 7, which is consistent with histological scoring of synovitis (Figure 3F). However, the signal is essentially undetectable beyond this time point, despite using long scans times, as the

myeloperoxidase/luminol system is substantially less sensitive compared to imaging using luciferase/luciferin.

Supplemental Figure 2

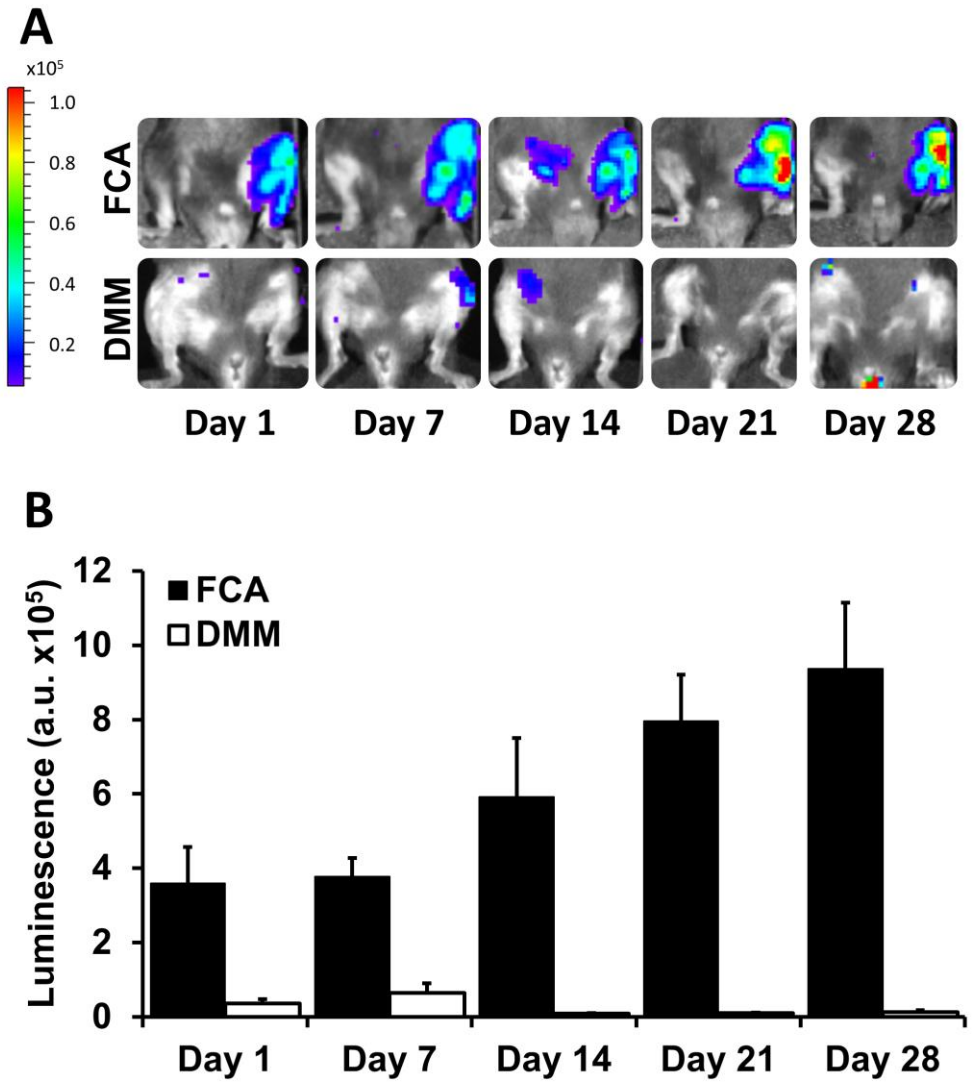

Supplemental Figure 3

Efficacy and endurance of *in vivo* expression of the target gene in the mouse knee joint was tested by intra-articular injection of 1.29 x 10^{10} gc of AAV-2/5 luciferase vector and monitored over time using the IVIS system. The luciferin (150mg/kg i.p.) kinetic curve was assessed at 7, 18, 26 and 36 days post-vector administration (**A**). Comparable magnitudes of response were noted up to day 26, with the 15 minute post luciferin injection time point being used for analysis in a group of 5 mice post DMM surgery. Representative IVIS scans taken at 14 (**B**) and 28 (**C**) days post-surgery appear comparable and analysis of luminescence (one way ANOVA) showed no significant difference over 28 days (data not shown). (**D**) Following intra-articular injection of AAV-2/5 lacZ vector (1.29 x 10^{10} gc), β-galactosidase expression (blue staining) in knee articular chondrocytes was still evident 21 days later, indicating cartilage matrix penetration. Scale bar = 20 μm. (**E**) Sections from mice transfected with the control vector (luciferase) or hPAR2 (with mCherry tag). Control mice show no immunoreactivity for mCherry whilst chondrocytes and synovial membrane in hPAR2-transfected mice show positive cellular staining. Note absence of mCherry staining in subchondral bone. Scale bars = 10 μm. Insets are isotype controls.

Supplemental Figure 3

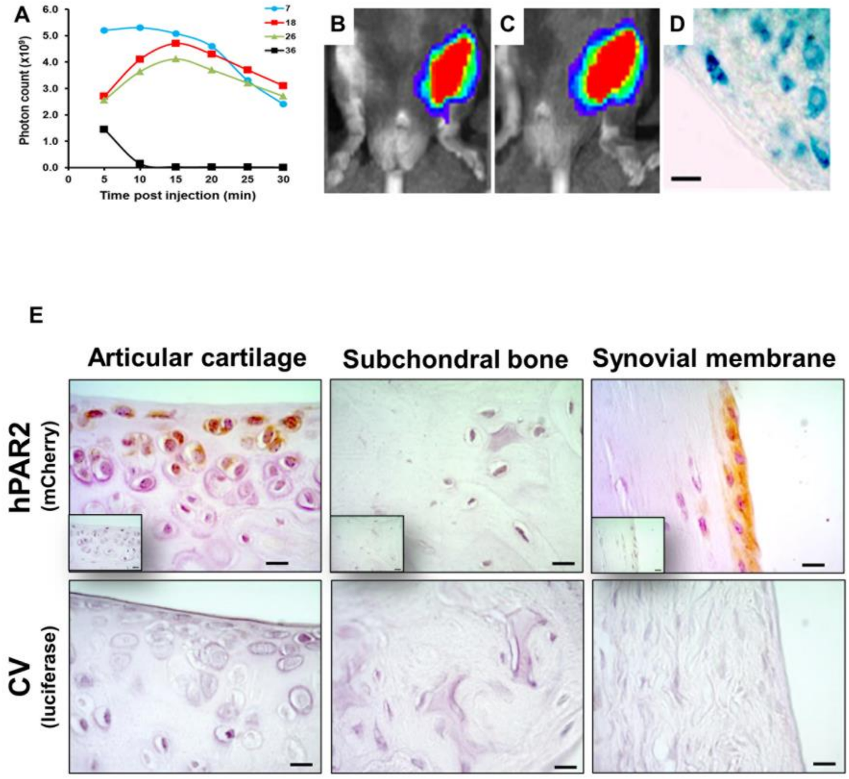

Supplemental Figure 4

Cartilage damage (A) and synovitis (B) scores comparing PAR2$^{+/+}$ (WT) with PAR2$^{-/-}$ mice (KO) and PAR2$^{-/-}$ mice administered control virus (CV) or those transfected with hPAR2. There was a significant difference in cartilage damage scores between PAR2 deficient mice administered hPAR2 compared to control virus and between WT and KO mice (* p = <0.02; ** p <0.01). There was no difference in these scores between CV and KO mice (P=0.04) nor between WT and hPAR2 mice (P=0.99).

There was no difference in synovitis scores between hPAR2 and CV mice (P= 0.29) and the difference between WT and KO mice just failed to achieve significance (P=0.057). There was no difference between CV and KO mice (P=0.8) nor between WT and hPAR2 mice (P=0.87).

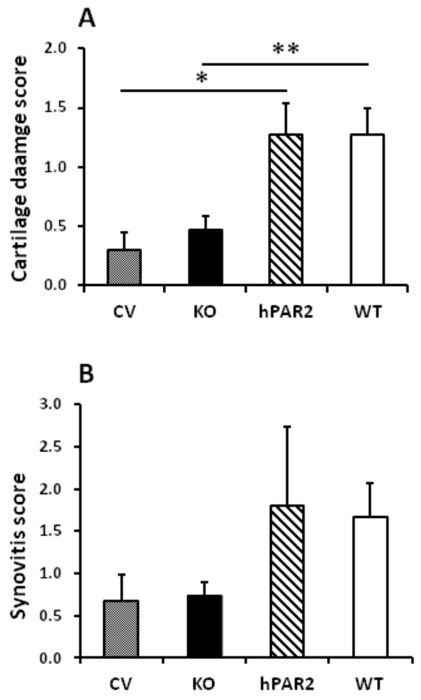

Supplemental Figure 5

The highly mineralised nature of osteophytes in $PAR2^{-/-}$ mice is indicative of a reduced proliferative chondrocytic phenotype.To evaluate a potential mechanistic explanation, we measured serum levels of the chondrocyte-associated miRNAs, let-7e and miR-140 in 14 week old mice. Notably, levels of Let-7e were significantly ($P<0.05$) lower in naive $PAR2^{-/-}$ mice compared to WT littermates (A) but miR-140 levels did not differ between genotype (B). It has been shown that suppression of let-7 induces a proliferation defect in chondrocytes via over-expression of *cdc34* and *E2F5* genes[1], which may explain why PAR2 deficiency is protective in the DMM model and tentatively suggests a potential molecular mechanism by which PAR2 influences OA pathogenesis.
