# Supplementary material for: Proteinase-activated receptor 2 modulates OA-related pain, cartilage and bone pathology
Source: Ann Rheum Dis. 2015 Dec 23;75(11):1989–97. doi: 10.1136/annrheumdis-2015-208268 (PMC5099200; doi:10.1136/annrheumdis-2015-208268)
Supplement: Web table [file annrheumdis-2015-208268-s4.pdf]

Supplemental Table 1. Synovitis Scoring

Score	Pannus	Synovial membrane thickening	Sub-synovial infiltration
**Description**	Defined as fibrous tissue/synovium/ inflammatory cell outgrowth spreading over the surface of the bone and/or cartilage at the joint margins.	Scored superior to the meniscal remnant. Score the maximum hyperplasia seen anywhere along this area.	Scored superior to the meniscal remnant.
**0**	No Pannus	1 cell thick	No infiltrating cells
**1**	Mild: Pannus has migrated on bone but not encroaching on cartilage.	Mild: 2-3 cells thick	Occasional scattered infiltrating cells or perivascular accumulations.
**2**	Moderate: Pannus has migrated < 1x cartilage depth.	Moderate: 4-5 cells thick	Focal areas of dense sub-synovial infiltrate, but still predominantly normal sub-synovial areolar connective tissue present.
**3**	Severe: Pannus has migrated > 1x cartilage depth.	Severe: > 6 cells thick	Widespread dense sub-synovial infiltrate – markedly reduced or little/no normal areolar connective tissue evident or lymphoid follicle formation.

(Modified from Jackson *et al*[6])
